# Vascular Endothelial Growth Factor and Its Soluble Receptor in Systemic Lupus Erythematosus Patients

**DOI:** 10.3390/biom12121884

**Published:** 2022-12-15

**Authors:** Fuensanta Gómez-Bernal, Yolanda Fernández-Cladera, Juan Carlos Quevedo-Abeledo, María García-González, Agustín F. González-Rivero, Antonia de Vera-González, Candelaria Martín-González, Miguel Á. González-Gay, Iván Ferraz-Amaro

**Affiliations:** 1 Division of Central Laboratory, Hospital Universitario de Canarias, 38320 Tenerife, Spain; 2 Division of Rheumatology, Hospital Doctor Negrín, 35010 Las Palmas de Gran Canaria, Spain; 3 Division of Rheumatology, Hospital Universitario de Canarias, 38320 Tenerife, Spain; 4 Division of Internal Medicine, Hospital Universitario de Canarias, 38320 Tenerife, Spain; 5 Department of Internal Medicine, University of La Laguna (ULL), 38200 Tenerife, Spain; 6 Epidemiology, Genetics and Atherosclerosis Research Group on Systemic Inflammatory Diseases, Hospital Universitario Marqués de Valdecilla, IDIVAL, 39011 Santander, Spain; 7 Division of Rheumatology, Hospital Universitario Marqués de Valdecilla, Universidad de Cantabria, 39011 Santander, Spain; 8 Cardiovascular Pathophysiology and Genomics Research Unit, School of Physiology, Faculty of Health Sciences, University of the Witwatersrand, Johannesburg 2000, South Africa

**Keywords:** vascular endothelial growth factor, systemic lupus erythematosus

## Abstract

Vascular endothelial growth factor (VEGF) is a major regulator of physiological and pathological angiogenesis. Its soluble receptor (sVEGFR) is a potent VEGF antagonist. Systemic lupus erythematosus (SLE) is an autoimmune disease with a diverse array of clinical manifestations that affect virtually any organ. We aimed to analyze the relationship of VEGF and sVEGFR with SLE disease-related features including disease activity, damage, and severity. Serum levels of VEGF165 isoform and sVEGFR (receptor 1) were assessed in 284 well-characterized patients with SLE. Linear regression analysis was performed to analyze the relationship of disease characteristics with both VEGF and sVEGFR. Patients with a disease damage index (SLICC score) equal to or greater than 1 had significantly elevated serum levels of VEGF and sVEGFR. Regarding disease-specific features, musculoskeletal manifestations were the disease feature most commonly associated with the upregulation of both VEGF and sVEGFR. SLE disease damage is associated with higher levels of VEGF and sVEGFR.

## 1. Introduction

Vascular endothelial growth factor (VEGF) is the dominant growth factor controlling angiogenesis in humans. VEGF is produced by a number of different cell types including diverse epithelial lineages, inflammatory and hematopoietic cells, and endothelial cells. It acts selectively on vascular endothelial cells, and is capable of stimulating angiogenesis in vitro and in vivo. Other direct actions of VEGF include stimulation of endothelial mitogenesis, promotion of endothelial survival, control of vascular permeability, increased expression of tissue plasminogen activator, urokinase plasminogen activator, collagenases, and matrix metalloproteinases [1,2]. For all these reasons, VEGF has well-recognized effects on various processes including, among others, lymphangiogenesis, metabolism, bone formation, hematopoiesis, and pathologic angiogenesis. [3]. Five different splicing variants of VEGF (VEGF_121_, VEGF_145_, VEGF_165_, VEGF_189_, and VEGF_206_) have been identified so far [4]. Several studies confirmed that native VEGF from numerous sources corresponds to VEGF_165_, the most diffusible isoform [5]. VEGFs mediate angiogenic signals to the vascular endothelium via high affinity receptor tyrosine kinases, designated soluble vascular endothelial growth factor receptor (sVEGFR). This is a potent VEGF antagonist, binding VEGF with high affinity [6]. Sufficient release of sVEGFR can be important in terms of prevention of exaggerated angiogenesis, and the VEGF/sVEGFR imbalance is crucial to the physiological homeostasis of vasculature and modulation of pro- and anti-angiogenesis.

Systemic lupus erythematosus (SLE) is a prototypic autoimmune disease characterized by the production of antibodies to components of the cell nucleus in association with a diverse array of clinical manifestations. The exact pathoetiology of SLE remains elusive. The multifactorial interaction between genetic and environmental factors together with hormonal factors influence the development of the disease. Defective immune regulatory mechanisms, such as those involved in the clearance of apoptotic cells and immune complexes, are also important in the pathogenesis of SLE [7].

In the present work we have assessed both VEGF and sVEGFR in a large series of well characterized patients with SLE. Our purpose was to analyze the relationship of VEGF and sVEGFR, and the relationship between them, with disease-related features, including disease activity, damage, and severity produced by this autoimmune disease.

## 2. Materials and Methods

### 2.1. Study Participants

Cross-sectional study that included 284 patients with SLE. All patients were 18 years old or older, had a clinical diagnosis of SLE, and fulfilled ≥4 American College of Rheumatology (ACR) classification criteria for SLE [8]. They had been diagnosed by rheumatologists and were periodically followed-up at rheumatology outpatient clinics. Patients taking prednisone, at an equivalent dose ≤10 mg/day, were allowed to participate, as glucocorticoids are often used in the treatment of SLE. The research was carried out in accordance with the Declaration of Helsinki. The study protocol was approved by the Institutional Review Committee at Hospital Universitario de Canarias and at Hospital Universitario Doctor Negrín (both in Spain), and all subjects provided informed written consent (Approval Number 2015_84).

### 2.2. Data Collection and Laboratory Assessments

Individuals included in the study completed a cardiovascular risk factor and medication use questionnaire and underwent a physical examination. Weight, height, body-mass index, abdominal circumference, and systolic and diastolic blood pressure (measured with the participant in a supine position) were assessed under standardized conditions. Information regarding smoking status (current smokers) and hypertension treatment was obtained from the questionnaire. Medical records were reviewed to ascertain specific diagnoses and medications. The Atherogenic index was calculated through Castelli’s formula: total cholesterol/HDL cholesterol. SLE disease activity and damage were assessed using the Systemic Lupus Erythematosus Disease Activity Index -2000 (SLEDAI-2K) [9] and the Systemic Lupus International Collaborating Clinics/American College of Rheumatology (SLICC/ACR Damage Index -SDI-) [10], respectively. For the propose of the present study, the SLEDAI-2k index was divided into none (0 points), mild (1–5 points), moderate (6–10 points), high (11–19), and very high activity (>20), as previously described [11]. Disease severity was also measured using the Katz Index [12]. An enzyme-linked immunosorbent assay (ELISA) kit was used for the detection of VEGF_165_ isoform and sVEGFR (receptor 1) (Elabscience, Houston, TX, USA). Both intra and inter-coefficients of variability were <10% for this assay. Carotid ultrasound to assess carotid intima-media wall thickness (cIMT) and presence of carotid plaques were assessed, as previously described [13].

### 2.3. Statistical Analysis

Demographic and clinical characteristics of patients were described as mean ± standard deviation (SD) or percentages for categorical variables. For non-normally distributed continuous variables, data were expressed as median and interquartile range (IQR). Relation of features of the disease with circulating VEGF and sVEGFR was assessed through linear regression analysis. All the analyses used a 5% two-sided significance level and were performed using Stata software, version 17/SE (StataCorp, College Station, TX, USA). *p*-values < 0.05 were considered statistically significant.

## 3. Results

### 3.1. Demographics and Disease-Related Data of Systemic Lupus Erythematosus Patients

Demographic and disease-related characteristics of the 284 patients with SLE included in this study are shown in Table 1. Most of them were women (92%), and the mean age ± SD was 50 ± 12 years. Body mass index of the participants was 28 ± 6 kg/m^2^, and the average abdominal circumference was 93 ± 14 cm. Classic cardiovascular risk factors were not uncommon. In this regard, 24% of the patients were current smokers, 39% had hypertension, and 30% were obese. Likewise, 25% of the patients were taking statins and 29% aspirin (Table 1).

Disease duration was 16 (IQR 7–24) years. Most of the patients were in the categories of no activity (40%) or mild-moderate activity (39%), as shown by the SLEDAI score. SLICC and Katz indexes were 1 (IQR 0–2) and 2 (IQR 1–4), respectively. Seventy-eight percent of the patients had a SLICC score equal to or higher than 1. Half of the patients were taking prednisone, with the median daily dose of prednisone being 5 mg/day (IQR 5–7.5 mg). At the time of recruitment, 67% patients were found to be positive for anti-DNA, and 69% were positive for ENA, with anti-SSA being the antibody most frequently found (35%). About two out of three patients (69%) were taking hydroxychloroquine when the study was performed. Other less commonly used disease-modifying antirheumatic drugs were methotrexate (11%) and azathioprine (15%). Additional information on SLE-related data is shown in Table 1.

VEGF serum levels in SLE patients were 176 (IQR 87-300) pg/mL, and circulating sVEGFR was 93 (IQR 54–184) pg/mL. Besides, the VEGF/sVEGFR ratio was 1.7 (IQR 0.8–3.5).

### 3.2. Demographics and Disease Characteristics Relation to VEGF and sVEGFR Serum Levels

Regarding demographics and cardiovascular comorbidity, only age and apolipoprotein A1 were the variables that showed significant association with sVEGFR and VEGF, respectively. In the case of cIMT and carotid plaque, no association was found with both molecules (Table 2). With regard to SLICC, when this score was considered as binary (equal or higher than 1), significant and positive relations to both VEGF and sVEGFR were disclosed. Besides, anti-SSA and anti-Sm, and IgM anticardiolipin antibodies, were associated with significantly higher values of VEGF. Regarding sVEGFR, while Katz index higher than 3 points was related to lower levels of this molecule, the use of prednisone was associated with significantly higher circulating levels of sVEGFR. Remarkably, demographics, cardiovascular comorbidity, subclinical carotid atherosclerosis, and SLE-related data were not associated with VEGF/sVEGFR ratio (Table 2).

### 3.3. Relationship of Activity Score, Damage and Disease Severity with VEGF and sVEGFR

Since disease scores represent the sum or combination of various aspects of the disease, we analyzed the relationship of their elements, one by one, with VEGF and sVEGFR (Table 3). Regarding the Katz index, the lowest hematocrit recorded to date of less than 30% was associated with lower VEGF levels. No other associations of the Katz index items were found with VEGF or sVEGFR. Regarding the SLEDAI score, which represents acute disease activity, only visual disturbance, present in a single patient, and pyuria, found in 11 subjects, were associated with significantly higher serum sVEGFR levels (Table 3). When evaluating SLICC, the only domain associated with significantly higher values of VEGF and sVEGFR was the musculoskeletal. Besides, the presence of malignancy was associated with significantly higher circulating sVEGFR but not VEGF levels. In the analysis of the full SLICC items (Supplementary Table S1) some other relationships were found. In this sense, retinal change or optic atrophy, and transverse myelitis, were related to higher circulating VEGF levels; and infarction or resection of bowel was associated with higher serum levels of sVEGFR. However, none of the score items correlated with the VEGF/sVEGFR ratio.

## 4. Discussion

The VEGF family is crucial in the regulation of angiogenesis, lymphangiogenesis, lipid metabolism, and inflammation. In the present study we observed that VEGF and sVEGFR are related to the damage caused by the disease in patients with SLE. This was especially true for the presence of musculoskeletal manifestations. 

In a previous study, serum VEGF levels were determined in 47 patients with SLE and 30 healthy controls [14]. In this study, serum VEGF levels were significantly higher in patients with active disease, as measured by the SLEDAI score. In addition, SLE patients with moderate and severe changes on nailfold capillaroscopy showed significantly higher serum VEGF levels than those with mild changes or healthy controls. These findings indicate that the serum level of VEGF may be a useful marker of disease activity and internal organ damage [14]. VEGF levels were also assessed in another study that included 84 women with SLE, 37 of them with antiphospholipid syndrome, along with 33 matched controls [15]. In this study, VEGF levels showed a statistically significant correlation with SLEDAI, and VEGF levels were also higher in anti-DNA positive patients. However, no association was found with antiphospholipid syndrome [15]. Our study included a larger series of patients and evaluated a number of disease-related manifestations, not only those associated with disease activity, but also damage and severity, cardiovascular comorbidity, or subclinical carotid atherosclerosis. Furthermore, in addition to serum VEGF levels, we also analyzed its receptor antagonist and the relationship between them, to better characterize the VEGF axis.

In our series the musculoskeletal manifestations of the disease were the ones that had a more consistent relationship with VEGF and sVEGFR. This finding may support the potential role of angiogenesis in the development of synovitis in general, not only related to SLE. In this sense, increased VEGF has been found in the synovial membrane, subchondral bone, synovial fluid, serum, and articular cartilage of patients with osteoarthritis [16]. Interestingly, serum and synovial VEGF concentrations are higher in patients with rheumatoid arthritis than in those with osteoarthritis or normal controls, and serum VEGF levels correlate with rheumatoid arthritis disease activity. In addition, VEGF has proinflammatory and antiapoptotic roles in the pathogenesis of rheumatoid arthritis, and induces tumor necrosis factor-α and interleukin 6 from mononuclear cells in the synovial fluid of patients with rheumatoid arthritis [17]. To our knowledge, our study is the first to specifically focus on the relationship between VEGF and joint disease in patients with SLE. Moreover, some relations were found between VEGF with anti-Sm, anti-SSA, and anti-ACA IgM. We believe that these autoantibodies could be expressing certain phenotypes of the disease with higher circulating VEGF.

The VEGF family is known to be involved in the development of atherosclerosis and other cardiovascular diseases. Furthermore, VEGFs have high potential as prognostic biomarkers, monitoring the progression and severity of cardiovascular diseases. Besides, scientific advances have led to the discovery of several VEGFs targeted experimental procedures for treating atherosclerosis [18]. In a previous study of 80 SLE patients, a significant correlation was found between cIMT and VEGF values. [19]. In addition, VEGF haplotypes were found to play a role in the development of severe ischemic manifestations in giant cell arteritis patients [20]. However, in our study we found no relationship between VEGF or sVEGFR with cIMT or carotid plaque in patients with SLE.

A major class of molecular targeted therapies has been designed to inhibit the VEGF axis. There are two main types of anti-VEGF drugs: targeting circulating VEGF and drugs interfering with the activity of the VEGF receptors. These therapies have proven effective in treating various types of solid cancers and eye diseases [21]. Given the findings presented in our work, we believe that clinical trials are needed to elucidate whether these therapies could be effective in SLE.

In our work we have analyzed VEGF_165_ because it is believed to be the most important isoform, which matches the native protein, and is more diffusible than other isoforms [5]. We also analyzed sVEGF receptor 1, known to be closely related to other types of receptors that share the same specific ligands [22]. However, we acknowledge the limitation that we cannot state that the findings of our study can be completely generalized to other isoforms of VEGF or other sVEGF receptors. We also recognize that the influence of treatments on VEGF and sVEGFR cannot be exactly known since our study has a cross-sectional design. Moreover, cumulative exposition to prednisone, hydroxychloroquine or other treatments was not assessed in our work. Besides, the role of VEGF/sVEGFR in the kidney disease of SLE patients could have been studied using immunohistochemistry in kidney samples. However, our study is fundamentally clinical, and we do not have biopsies from patients with lupus nephritis, an important consideration for future studies. 

## 5. Conclusions

In conclusion, VEGF family is associated with several clinical characteristics of patients with SLE. VEGF and sVEGFR may play a role in the pathophysiology of the disease.

## Figures and Tables

**Table 1:** Characteristics of SLE patients.

		SLE Patients
		(n = 284)
Age, years		50 ± 12
Female, n (%)		261 (92)
Body mass index, kg/m^2^		28 ± 6
Abdominal circumference, cm		93 ± 14
Hip circumference, cm		103 ± 12
Waist-to-hip ratio		0.90 ± 0.07
Vascular Endothelial Growth Factor		
	VEGF, pg/mL	176 (87–300)
	sVEGFR, pg/mL	93 (54–184)
	Ratio VEGF/sVEGFR	1.7 (0.8–3.5)
Cardiovascular co-morbidity		
Smoking, n (%)		69 (24)
Diabetes, n (%)		16 (6)
Hypertension, n (%)		111 (39)
Obesity, n (%)		85 (30)
Statins, n (%)		72 (25)
Aspirin, n (%)		80 (29)
Lipid profile		
	Cholesterol, mg/dL	198 ± 36
	Triglycerides, mg/dL	130 ± 78
	LDL cholesterol, mg/dL	111 ± 29
	HDL cholesterol, mg/dL	61 ± 19
	LDL:HDL cholesterol ratio	1.96 ± 0.75
	Non-HDL cholesterol, mg/dL	137 ± 33
	Lipoprotein (a), mg/dL	39 (12–108)
	Apolipoprotein A1, mg/dL	173 ± 35
	Apolipoprotein B, mg/dL	95 ± 23
	Apo B:Apo A1 ratio	0.57 ± 0.17
	Atherogenic index	3.5 ± 1.1
Carotid intima media thickness, microns		628 ± 109
Carotid plaque, n (%)		99 (36)
SLE related data		
Disease duration, years		16 (7–24)
CRP, mg/dl		2.0 (0.8–4.4)
SLICC		1 (0–2)
SLICC ≥1, n (%)		191 (68)
Katz Index		2 (1–4)
Katz ≥3, n (%)		126 (44)
SLEDAI		2 (0–4)
SLEDAI categories, n (%)		
	No activity, n (%)	109 (40)
	Mild, n (%)	107 (39)
	Moderate, n (%)	41 (15)
	High, n (%)	10 (4)
	Very High, n (%)	4 (1)
Auto-antibody profile		
	Anti-DNA positive, n (%)	151 (67)
	ENA positive, n (%)	164 (69)
	Anti-SSA, n (%)	55 (35)
	Anti-SSB, n (%)	36 (21)
	Anti-RNP, n (%)	64 (28)
	Anti-Sm, n (%)	24 (10)
	Anti-ribosome	13 (9)
	Anti-nucleosome	32 (22)
	Anti-histone	22 (15)
Antiphospholipid syndrome, n (%)		43 (16)
Antiphospholipid autoantibodies, n (%)		61 (32)
	Lupus anticoagulant, n (%)	51 (28)
	ACA IgM, n (%)	22 (11)
	ACA IgG, n (%)	39 (20)
	Anti beta2 glycoprotein IgM, n (%)	19 (10)
	Anti beta2 glycoprotein IgG, n (%)	28 (15)
C3, mg/dL		130 ± 40
C4, mg/dL		21 ± 12
Current prednisone, n (%)		140 (50)
Prednisone, mg/day		5 (5–7.5)
Hydroxychloroquine, n (%)		194 (69)
Methotrexate, n (%)		31 (11)
Mycophenolate mofetil, n (%)		31 (11)
Azathioprine, n (%)		43 (15)
Rituximab, n (%)		8 (3)
Belimumab, n (%)		8 (3)

Data represent mean ± SD or median (interquartile range) when data were not normally distributed. BMI: body mass index; C3 C4: complement; CRP: C reactive protein; LDL: low-density lipoprotein. DMARD: disease-modifying antirheumatic drug; ACA: anticardiolipin. HDL: high-density lipoprotein; ANA: antinuclear antibodies; ENA: extractible nuclear antibodies. SLEDAI: Systemic Lupus Erythematosus Disease Activity Index. SLEDAI categories were defined as: 0, no activity; 1–5 mild; 6–10 moderate; >10 high activity, >20 very high activity. SLICC: Systemic Lupus International Collaborating Clinics/American Colleague of Rheumatology Damage Index. VEGF: Vascular Endothelial Growth Factor; sVEGFR: soluble Vascular Endothelial Growth Factor receptor.

**Table 2:** Demographics and disease characteristics relation to VEGF and sVEGFR serum levels.

		*log* VEGF x100, pg/mL		sVEGFR, pg/mL		Ratio VEGF/sVEGFR
		Beta Coef. (95%), *p*				*p*
Age, years		0.7 (−0.3–2)	0.16	2 (1–3)	0.004	0.35
Female		24 (−17–65)	0.25	4 (−45–52)	0.87	0.20
Body mass index, kg/m^2^		−0.8 (−3–1)	0.43	0.03 (−2–2)	0.97	0.55
Abdominal circumference, cm		−0.2 (−1–0.7)	0.68	1 (−0.4–2)	0.26	0.40
Hip circumference, cm		−0.4 (−1–0.6)	0.47	0.3 (−1–2)	0.56	0.41
Waist-to-hip ratio		−54 (−113–5)	0.070	103 (−78–285)	0.26	0.80
Cardiovascular co-morbidity						
Smoking		9 (−17–35)	0.50	−2 (−33–29)	0.91	0.65
Diabetes		24 (−23–72)	0.32	46 (−11–103)	0.11	0.88
Hypertension		11 (−12–34)	0.35	2 (−26–29)	0.90	0.32
Obesity		6 (−19–30)	0.64	7 (−23–36)	0.66	0.11
Statins		20 (−5–46)	0.12	−3 (−34–28)	0.85	0.25
Aspirin		−5 (−30–20)	0.67	−3 (−33–28)	0.86	0.88
Lipid profile						
	Cholesterol, mg/dL	0.3 (−0.02–0.6)	0.069	0.3 (−0.02–0.7)	0.069	0.26
	Triglycerides, mg/dL	0.1 (−0.03–0.03)	0.12	0.1 (−0.05–0.3)	0.15	0.69
	LDL cholesterol, mg/dL	0.08 (−0.3–0.5)	0.69	0.3 (−0.1–0.8)	0.17	0.58
	HDL cholesterol, mg/dL	0.5 (−0.1–1)	0.11	0.1 (−0.6–0.8)	0.82	0.32
	LDL:HDL cholesterol ratio	−2 (−17–14)	0.83	10 (−8–28)	0.29	0.56
	Non-HDL cholesterol, mg/dL	0.2 (−0.2–0.5)	0.29	0.4 (−0.02–1)	0.061	0.50
	Lipoprotein (a), mg/dL	0.1 (−0.02–0.2)	0.11	−0.003 (−0.12–0.14)	0.97	0.65
	Apolipoprotein A1, mg/dL	**0.4 (0.1–0.8)**	**0.006**	0.3 (−0.1–0.6)	0.20	0.30
	Apolipoprotein B, mg/dL	0.5 (−0.02–1)	0.058	0.4 (−0.2–1)	0.22	0.19
	Apo B:Apo A1 ratio	3 (−65–70)	0.94	12 (−70–95)	0.77	0.78
	Atherogenic index	3 (−8–13)	0.60	8 (−5–21)	0.21	0.63
cIMT, microns		0.06 (−0.05–0.2)	0.28	0.1 (−0.04–0.2)	0.19	0.91
Carotid plaque		1 (−23–25)	0.94	18 (−10–47)	0.21	0.70
SLE related data						
Disease duration, years		0.5 (−0.7–2)	0.42	1 (−0.3–2)	0.13	0.26
CRP, mg/dL		0.7 (−0.3–2)	0.17	0.4 (−0.8–2)	0.49	0.64
SLICC		5 (−1–12)	0.11	5 (−2–13)	0.17	0.63
SLICC ≥1		**26 (2–50)**	**0.033**	**46 (18–74)**	**0.001**	0.43
Katz Index		−0.4 (−6–5)	0.88	−5 (−12–2)	0.15	0.72
Katz ≥3		−9 (−32–14)	0.44	**−29 (−56– (−3))**	**0.032**	0.83
SLEDAI		−0.8 (−4–2)	0.57	0.4 (−3–4)	0.80	0.18
SLEDAI categories						
	No activity	ref.		ref.		ref.
	Mild	−1 (−27–25)	0.93	21 (−10–51)	0.19	0.37
	Moderate to very high	0.5 (−31–32)	0.97	29 (−9–67)	0.14	0.080
Auto-antibody profile						
	Anti-DNA positive	20 (−8–47)	0.16	−12 (−43–20)	0.46	0.69
	ENA positive	−0.06 (−26–26)	0.99	−15 (−56–26)	0.47	0.35
	Anti-SSA	**39 (7–71)**	**0.017**	−30 (−123–64)	0.53	0.51
	Anti-SSB	63 (−10–136)	0.089	−1 (−34–33)	0.97	0.32
	Anti-RNP	14 (−14–41)	0.33	−8 (−56–41)	0.75	0.88
	Anti-Sm	**47 (8–86)**	**0.018**	38 (−33–108)	0.30	0.49
	Anti-ribosome	−14 (−70–41)	0.61	−42 (−93–8)	0.10	0.41
	Anti-nucleosome	−2 (−41–37)	0.92	−42 (−99–16)	0.15	0.11
	Anti-histone	−6 (−51–39)	0.79	34 (−1–69)	0.059	0.98
Antiphospholipid syndrome		−8 (−41–24)	0.61	−12 (−50–26)	0.53	0.87
Antiphospholipid autoantibodies						
	Lupus anticoagulant	−15 (−42–13)	0.29	−32 (−69–5)	0.093	0.45
	ACA IgM	**47 (7–87)**	**0.021**	26 (−24–77)	0.30	0.084
	ACA IgG	23 (−8–54)	0.14	35 (−4–75)	0.078	0.26
	Anti beta2 glycoprotein IgM	−15 (−59–30)	0.51	0.3 (−56–57)	0.99	0.14
	Anti beta2 glycoprotein IgG	7 (−28–42)	0.70	41 (−5–88)	0.079	0.61
C3, mg/dL		0.02 (−0.3–0.3)	0.87	−0.002 (−0.4–0.4)	0.99	0.32
C4, mg/dL		0.3 (−0.7–1)	0.57	−1 (−2–1)	0.27	0.11
Current prednisone		20 (−2–43)	0.079	−12 (−39–15)	0.40	0.32
Prednisone, mg/day		2 (−3–7)	0.48	**7 (1–13)**	**0.027**	0.48
Hydroxychloroquine		−9 (−33–16)	0.49	3 (−220–225)	0.98	0.86
Methotrexate		−27 (−64–9)	0.14	2 (−41–45)	0.93	0.25
Mycophenolate mofetil		−24 (−60–12)	0.20	−42 (−85–1)	0.058	0.69
Azathioprine		−2 (−33–29)	0.89	9 (−29–47)	0.63	0.43
Rituximab		−16 (−82–51)	0.64	−60 (−140–19)	0.13	0.89
Belimumab		−44 (−110–22)	0.19	−73 (−152–7)	0.071	0.86

In this analysis VEGF, sVEGFR, and ratio were considered the dependent variables. For VEGF/sVEGFR ratio, beta coefficients are not shown. Significant *p* values are depicted in bold. BMI: body mass index; C3 C4: complement; CRP: C reactive protein; LDL: low-density lipoprotein. DMARD: disease-modifying antirheumatic drug; ACA: anticardiolipin; cIMT: carotid intima thickness. HDL: high-density lipoprotein; ANA: antinuclear antibodies; ENA: extractible nuclear antibodies. SLEDAI: Systemic Lupus Erythematosus Disease Activity Index. SLEDAI categories were defined as: 0, no activity; 1–5 mild; 6–10 moderate; >10 high activity, >20 very high activity. SLICC: Systemic Lupus International Collaborating Clinics/American Colleague of Rheumatology Damage Index. VEGF: Vascular Endothelial Growth Factor; sVEGFR: soluble Vascular Endothelial Growth Factor receptor.

**Table 3:** Individual disease score items related to VEGF and sVEGFR serum levels.

			*log* VEGF × 100, pg/mL		sVEGFR, pg/mL		Ratio VEGF/sVEGFR
	n	%	Beta Coef. (95%)	*p*	Beta Coef. (95%)	*p*	*p*
Katz index							
History of cerebritis (seizure or organic brain syndrome)	12	6	17 (−14–48)	0.28	−7 (−42–28)	0.69	0.14
History of pulmonary disease	10	5	4 (−30–39)	0.80	3 (−38–44)	0.89	0.84
Biopsy proven diffuse proliferative glomerulonephritis	23	12	11 (−11–32)	0.32	6 (−21–33)	0.66	0.88
4–6 ARA criteria for SLE satisfied to date	139	73	9 (−24–41)	0.60	13 (−27–52)	0.53	0.59
7 or more ARA criteria for SLE satisfied to date	23	12	−4 (−26–18)	0.74	−4 (−30–23)	0.79	0.33
History of proteinuria (2+ or more)	62	32	−2 (−33–28)	0.89	−32 (−70–6)	0.095	0.20
Lowest recorded hematocrit to date = 30–37%	88	46	**29 (0.3–57)**	**0.047**	−2 (−38–33)	0.90	0.14
Lowest recorded hematocrit to date <30%	47	25	**−26 (−42–(−9))**	**0.002**	−20 (−40–1)	0.056	0.16
Highest recorded creatinine to date = 1.3–3	28	15	14 (−27–56)	0.49	−26 (−75–24)	0.30	0.31
Highest recorded creatinine to date >3	3	2	19 (−3–75)	0.50	−4 (−73–66)	0.92	0.86
SLEDAI							
Seizures	1	0	97 (−89–283)	0.31	70 (−154–295)	0.54	0.81
Psychosis	1	0	89 (−97–275)	0.35	77 (−147–302)	0.50	0.76
Organic brain syndrome	0	0	-	-	-	-	-
Visual disturbance	1	0	28 (−158–215)	0.77	**309 (88–531)**	**0.006**	0.50
Cranial nerve disorder	1	0	−6 (−193–180)	0.95	−23 (−248–202)	0.84	0.67
Lupus headache	1	0	114 (−72–300)	0.23	9 (−216–234)	0.94	0.89
ACVA	0	0	-	-	-	-	-
Vasculitis	1	0	140 (−46–326)	0.14	214 (−9–437)	0.060	0.76
Arthritis	9	3	−30 (−93–33)	0.35	−0.14 (−76–76)	0.99	0.16
Myositis	0	0	-	-	-	-	-
Urinary cylinders	7	3	21 (−51–92)	0.57	30 (−56–116)	0.49	0.36
Hematuria	16	6	29 (−19–77)	0.24	14 (−44–72)	0.63	0.47
Proteinuria	5	2	−35 (−119–49)	0.41	51 (−50–153)	0.32	0.46
Pyuria	11	4	48 (−9–105)	0.099	**104 (36–172)**	**0.003**	0.38
Rash	21	8	28 (−15–70)	0.20	2 (−49–53)	0.95	0.71
Alopecia	11	4	−5 (−62–53)	0.87	−2 (−71–67)	0.95	0.41
Mucosal ulcers	14	5	−42 (−93–9)	0.10	37 (−24–98)	0.24	0.16
Pleurisy	3	1	−48 (−156–60)	0.38	−51 (−181–79)	0.44	0.53
Pericarditis	1	0	40 (−147–227)	0.67	53 (−171–278)	0.64	0.65
Low complement	76	28	1 (−24–27)	0.92	−11 (−42–21)	0.51	0.65
Elevated antiDNA	85	31	−6 (−31–19)	0.66	−7 (−37–23)	0.65	0.97
Fever	2	1	17 (−149–116)	0.80	158 (−0.14–316)	0.050	0.35
Thrombopenia	10	4	19 (−41–79)	0.53	60 (−12–132)	0.10	0.89
Leukopenia	19	7	20 (−26–65)	0.39	23 (−30–77)	0.39	0.42
SLICC domains							
Ocular	63	22	−16 (−43–11)	0.26	−10 (−42–22)	0.56	0.95
Neuropsychiatric	40	14	17 (−15–49)	0.30	22 (−17–60)	0.27	0.78
Renal	28	10	18 (−19–56)	0.34	17 (−28–62)	0.45	0.63
Pulmonary	19	7	−12 (−56–32)	0.58	−24 (−83–35)	0.43	0.67
Cardiovascular	23	8	11 (−30–52)	0.60	−10 (−58–39)	0.70	0.77
Peripheral vascular	34	12	11 (−24–45)	0.54	4 (−37–45)	0.84	0.68
Gastrointestinal	28	10	21 (−16–57)	0.27	44 (−0.8–89)	0.054	0.52
Musculoskeletal	89	31	**37 (14–61)**	**0.002**	**43 (14–71)**	**0.003**	0.78
Skin	39	14	3 (−9–15)	0.21	−8 (−47–30)	0.67	0.34
Premature gonadal failure	19	7	20 (−25–65)	0.38	18 (−35–71)	0.50	0.65
Diabetes (regardless of treatment)	18	6	22 (−23–67)	0.33	36 (−18–90)	0.19	0.80
Malignancy (excluded dysplasia)	11	4	−42 (−98–15)	0.15	79 (11–146)	0.023	0.39

History of pulmonary disease refers to the presence of lupus pneumonitis, pulmonary hemorrhage or pulmonary hypertension. SLEDAI: Systemic Lupus Erythematosus Disease Activity Index; SLE: Systemic Lupus Erythematosus. SLICC: Systemic Lupus International Collaborating Clinics/American Colleague of Rheumatology Damage Index. The presence of a SLICC domain involvement is shown if points in the domain are ≥1. See Supplementary Table S1. ARA: American Rheumatism Association; ACVA: Acute Cerebrovascular Accident. Significant *p* values are depicted in bold.

## Data Availability

The data sets used and/or analyzed in the present study are available from the corresponding author upon request.

## Supplementary Materials

The following supporting information can be downloaded at: https://www.mdpi.com/article/10.3390/biom12121884/s1, Supplementary Table S1. Relation of SLICC score items to VEGF axis.
