# Supplementary material for: Vascular Endothelial Growth Factor and Its Soluble Receptor in Systemic Lupus Erythematosus Patients
Source: Biomolecules. 2022 Dec 15;12(12):1884. doi: 10.3390/biom12121884 (PMC9775345; doi:10.3390/biom12121884)
Supplement: Supplementary file 1 [file biomolecules-12-01884-s001.zip › biomolecules-2052101-supplementary.pdf]

Supplementary Table S1. Relation of SLICC score items to VEGF axis.

			log VEGF x 100, pg/ml		sVEGFR, pg/ml		ratio VEGF/sVEGF
	n	%	beta coef. (95%)	*p*	beta coef. (95%)	*p*	*p*
Ocular							
Any cataract ever	29	11	-9 (-47-30)	0.66	-25 (-70-19)	0.27	0.11
Retinal change or optic atrophy	33	12	**-46 (-81- -10)**	**0.011**	-28 (-69-14)	0.19	0.37
Points =>1 in the domain	63	22	-16 (-43-11)	0.26	-10 (-42-22)	0.56	0.95
Neuropsychiatric							
Cognitive impairment	7	3	42 (-28-113)	0.24	74 (-11-159)	0.088	0.73
Seizures requiring therapy for 6 months	15	5	22 (-27-71)	0.38	19 (-40-78)	0.53	0.85
Cerebrovascular accident ever	13	5	8 (-46-63)	0.76	-48 (-160-64)	0.40	0.95
Cranial or peripheral neuropathy	5	2	-3 (-96-90)	0.95	-48 (-160-64)	0.40	0.75
Transverse myelitis	1	0	**-191 (-375- -7)**	**0.042**	-126 (-349-97)	0.27	0.90
Points =>1 in the domain	40	14	17 (-15-49)	0.30	22 (-17-60)	0.27	0.78
Renal							
Estimated or measured glomerular filtration rate <50%	13	5	-15 (-70-40)	0.59	-9 (-74-57)	0.80	0.36
Proteinuria 3.5 gm/24 hours	7	3	70 (-0,1-141)	0.050	48 (-37-133)	0.26	0.80
End-stage renal disease	4	1	25 (-6-56)	0.11	-13 (-51-24)	0.49	0.15
Points =>1 in the domain	28	10	18 (-19-56)	0.34	17 (-28-62)	0.45	0.63
Pulmonary							
Pulmonary hypertension	4	1	-35 (-128-58)	0.46	-0.5 (-113-112)	0.99	0.59
Pulmonary fibrosis	4	1	33 (-61-126)	0.49	-19 (-132-93)	0.74	0.95
Shrinking lung	2	1	-27 (-158-105)	0.69	-58 (-217-100)	0.47	0.54
Pleural fibrosis	1	0	7 (-178-193)	095	-89 (-313-135)	0.43	0.80
Pulmonary infarction	1	0	-45 (-231-141)	0.64	-42 (-266-183)	0.71	0.62
Points =>1 in the domain	19	7	-12 (-56-32)	0.58	-24 (-83-35)	0.43	0.67
Cardiovascular							
Angina or coronary artery bypass	4	1	16 (-77-109)	0.74	76 (-36-189)	0.18	0.42
Myocardial infarction ever	2	1	-4 (-135-128)	0.96	56 (-103-215)	0.49	0.50
Cardiomyopathy	2	1	-80 (-211-51)	0.23	-61 (-220-97)	0.45	0.82
Valvular disease	9	3	18 (-48-85)	0.59	-32 (-107-44)	0.41	0.31
Pericarditis for 6 months, or pericardiectomy	2	1	87 (-44-218)	0.19	-2 (-162-157)	0.98	0.70
Points =>1 in the domain	23	8	11 (-30-52)	0.60	-10 (-58-39)	0.70	0.77
Peripheral vascular							
Claudication for 6 months	3	1	26 (-82-134)	0.64	66 (-64-195)	0.32	0.41
Minor tissue loss (pulp space)	5	2	33 (-50-117)	0.43	-45 (-146-56)	0.38	**0.016**

Significant tissue loss ever	0	0	-	-	-	-	-
Venous thrombosis	14	5	-23 (-76-30)	0.39	-21 (-83-40)	0.49	0.17
Points =>1 in the domain	34	12	11 (-24-45)	0.54	4 (-37-45)	0.84	0.68
Gastrointestinal							
Infarction or resection of terminal duodenum, spleen, liver or gallbladder for any reason	22	8	22 (-19-63)	0.29	**53 (2-103)**	**0.040**	0.46
Mesenteric insufficiency	1	0	-49 (-234-136)	0.60	-87 (-310-137)	0.45	0.82
Chronic peritonitis	1	0	138(-47-322)	0.14	102 (-121-325)	0.37	0.94
Stricture or upper gastrointestinal tract surgery ever	0	0	-	-	-	-	-
Pancreatic insufficiency	0	0	-	-	-	-	-
Points =>1 in the domain	28	10	21 (-16-57)	0.27	44 (-0.8-89)	0.054	0.52
Musculoskeletal							
Muscle atrophy or weakness	3	1	46 (-63-154)	0.41	47 (-85-178)	0.49	0.74
Deforming or erosive arthritis	40	15	**41 (9-73)**	**0.013**	**40 (1-79)**	**0.042**	0.69
Osteoporosis with fracture or vertebral collapse	23	9	25 (-16-67)	0.23	**57 (8-106)**	**0.023**	0.46
Avascular necrosis	7	3	29 (-43-101)	0.42	-69 (-155-17)	0.12	0.11
Osteomyelitis	1	0	29 (-158-216)	0.76	**309 (86-533)**	**0.007**	0.50
Tendon rupture	4	2	27 (-67-121)	0.57	74 (-40-187)	0.20	0.73
Points =>1 in the domain	89	31	**37 (14-61)**	**0.002**	**43 (14-71)**	**0.003**	0.78
Skin							
Scarring chronic alopecia	16	6	-5 (-53-42)	0.82	-33 (-91-25)	0.27	0.43
Extensive scarring or panniculitis	10	4	3 (-57-63)	0.92	26 (-46-99)	0.48	0.50
Skin ulceration	4	1	-11 (-118-97)	0.85	-75 (-188-38)	0.19	0.86
Points =>1 in the domain	39	14	3 (-9-15)	0.21	-8 (-47-30)	0.67	0.34
Premature gonadal failure	19	7	20 (-25-65)	0.38	18 (-35-71)	0.50	0.65
Diabetes (regardless of treatment)	18	6	22 (-23-67)	0.33	36 (-18-90)	0.19	0.80
Malignancy (exclude dysplasia)	11	4	-42 (-98-15)	0.15	**79 (11-146)**	**0.023**	0.39

SLICC items and domains represent the independent variable. Significant p values are depicted in bold.

SLICC: Systemic Lupus International Collaborating Clinics/American College of Rheumatology Damage Index.
